# Spontaneous activity in whisker-innervating region of neonatal mouse trigeminal ganglion

**DOI:** 10.1038/s41598-022-20068-z

**Published:** 2022-09-29

**Authors:** Piu Banerjee, Fumi Kubo, Hirofumi Nakaoka, Rieko Ajima, Takuya Sato, Tatsumi Hirata, Takuji Iwasato

**Affiliations:** 1 Laboratory of Mammalian Neural Circuits, National Institute of Genetics (NIG), Mishima, Japan; 2 Department of Genetics, SOKENDAI, Mishima, Japan; 3 Laboratory of Systems Neuroscience, NIG, Mishima, Japan; 4 Department of Cancer Genome Research, Sasaki Institute, Tokyo, Japan; 5 Laboratory of Mammalian Development, NIG, Mishima, Japan; 6 Laboratory of Brain Function, NIG, Mishima, Japan

**Keywords:** Development of the nervous system, Neural circuits, Somatosensory system

## Abstract

Spontaneous activity during the early postnatal period is thought to be crucial for the establishment of mature neural circuits. It remains unclear if the peripheral structure of the developing somatosensory system exhibits spontaneous activity, similar to that observed in the retina and cochlea of developing mammals. By establishing an ex vivo calcium imaging system, here we found that neurons in the whisker-innervating region of the trigeminal ganglion (TG) of neonatal mice generate spontaneous activity. A small percentage of neurons showed some obvious correlated activity, and these neurons were mostly located close to one another. TG spontaneous activity was majorly exhibited by medium-to-large diameter neurons, a characteristic of mechanosensory neurons, and was blocked by chelation of extracellular calcium. Moreover, this activity was diminished by the adult stage. Spontaneous activity in the TG during the first postnatal week could be a source of spontaneous activity observed in the neonatal mouse barrel cortex.

## Introduction

Studying the construction of mature neural circuits is crucial to comprehending their complexities. In the embryonic stage, genetic factors contribute to laying down the coarse neural networks, which are then refined postnatally by neuronal activity. The mammalian sensory cortices show correlated spontaneous activity during the early postnatal period^1–8^. Accumulating evidence suggests that such activity plays an important role in the refinement of immature neural circuits following the Hebbian principles of plasticity^9–11^.

Among the developing sensory cortices, spontaneous activity was first identified in the mammalian visual cortex. In the visual cortex, spontaneous activity is observed during the early postnatal period, which propagates tangentially in a wave-like manner^4,12^. This activity originates from the retina, wherein the wave-type spontaneous activity was first discovered in vitro^4,13–17^. Similarly, in the auditory cortex, spontaneous activity is observed during the early postnatal period, which resembles the future tonotopic axis^18^. This activity originates from the cochlea, wherein spontaneous bursts of action potentials were first found ex vivo^6,10,19–25^. Hence, in the developing visual and auditory systems, the spontaneous activity in the cortices originates from their respective peripheral structures. In the somatosensory cortex, spontaneous activity is observed during the first postnatal week^5,7,8,26–30^, whose pattern corresponds to the barrel map, thereby creating a patchwork-like appearance^5^. In patchwork-type activity, the neurons within the same barrel fire together, therefore indicating the likelihood of this activity being crucial for the formation of a precise somatotopic map^5,7^. However, a paucity exists in our understanding of the source of the patchwork-type spontaneous activity. Some studies suggest that prior to the onset of exploratory whisker movement, sensory feedback from inadvertent whisker twitches is the source of spontaneous activity in the somatosensory cortex^31–34^. Contrary to this, recent work by our group demonstrated that only a small percentage (~ 11%) of patchwork-type spontaneous activity correlates with whisker twitches^5^. Hence, the major source of patchwork-type spontaneous activity remains unknown. Furthermore, to our knowledge, no study has elucidated whether the peripheral structure of the developing somatosensory system exhibits spontaneous activity itself, as is the case in visual and auditory systems.

Our previous study revealed that the patchwork-type spontaneous activity observed in the neonatal mouse barrel cortex is blocked by the injection of a local anesthetic to the contralateral whisker pad but not by transection of the infraorbital nerve (ION)^5^, implying that the source is somewhere in the periphery but downstream of the ION. Based on these results, we postulated that the trigeminal ganglion (TG), which harbors the cell bodies of neurons that innervate the whisker pad, is the source of patchwork-type spontaneous activity in the somatosensory cortex. In the present study, we aim to elucidate whether the neonatal mouse TG exhibits spontaneous activity itself. To eliminate all inputs from the whisker pad while simultaneously preserving the cytoarchitecture of the TG, we established a novel ex vivo calcium imaging system to delineate the occurrence of spontaneous firing by neonatal TG neurons. With this method, we discovered that neurons in the whisker innervating area of the mouse TG generate spontaneous activity during the early postnatal period, but this activity largely diminishes by adulthood. The bulk of the spontaneously firing neurons in the neonatal stage have a medium-to-large diameter, which is a characteristic of the mechanosensory neurons. The spontaneous activity in neonatal TG is sporadic, with no obvious spatiotemporal pattern or wide-scale co-activation of neurons. Nevertheless, a small population of neuron pairs, majorly located close to one another shows some evident correlation. The present study is the first to show that spontaneous activity is found in the peripheral structure of the somatosensory system during the neonatal stage.

## Results

### An ex vivo imaging system for neonatal TG

**Figure 1:**
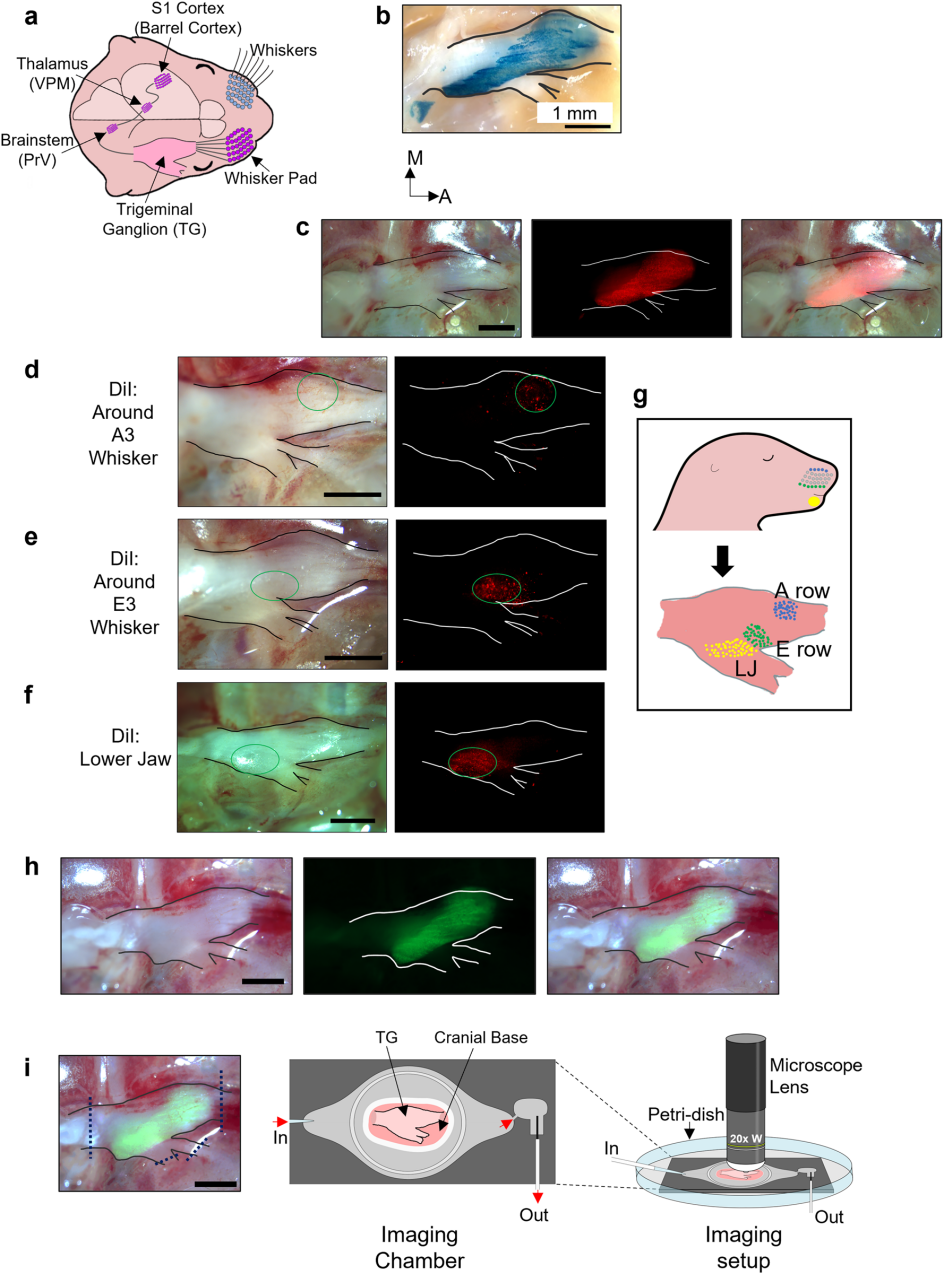
Identification of whisker-innervating region in intact TG ex vivo. (**a**) Illustration of the mouse whisker-barrel system. (**b**) Representative image along the dorsal surface of an intact TG from *Avil*-Cre:RNZ mouse at P6. The spatial distribution of peripheral sensory neurons is visualized by X-Gal staining of the whole TG (P6; N = 4 mice). TG outline is shown. M: medial, A: anterior. (**c**) Representative image of an intact TG from *Avil*-nlsRFP mouse at P5. Brightfield (Left), RFP (Middle), Merged (Right) images. (P4–P6; N = 4). (**d**–**f**) Labeled neuron localization in the P5 TG 5 days after placing DiI crystals around A3 whisker (N = 3) (**d**), around E3 whisker (N = 3) (**e**) and lower jaw (N = 3) (**f**). Brightfield image (Left) and RFP filter image (Right) are shown. Green circles enclose the region of labeled neuron localization. (**g**) Schematic showing the whisker-row-dependent topography along the dorsal surface of the intact TG. (**h**) Representative image of the intact TG from the *Avil*-Cre:*R26*-GCaMP6s mouse at P5. Brightfield image (Left), GCaMP6s image (Middle), Merged image (Right). (**i**) (Left) Dashed lines indicates where the TG is transected from its peripheral and central connections. (Middle) Schematic of the imaging chamber used for ex vivo imaging. An intact TG attached to the cranial base is glued to the base of the chamber. (Right) Schematic of the ex vivo imaging setup. The imaging chamber is fixed to the bottom of a 10 cm-diameter petri-dish. The chamber is constantly perfused with buffers and a 20X water immersion lens is used for imaging. Scale bars: 1 mm.

Input from each whisker goes to TG, the principal trigeminal nucleus (PrV) in the brainstem, the ventral posteromedial nucleus (VPM) in the thalamus, and finally reaches barrel cortex layer 4, where each barrel receives input from a single whisker (Fig. 1a). The TG has pseudo-unipolar sensory neurons whose processes innervate the periphery and brainstem. To identify the location of sensory neurons in the whole TG, we crossed the *Avil*-Cre mouse, which expresses Cre recombinase in peripheral sensory neurons, with the *Rosa26 (R26)*-loxP-stop-loxP (LSL)-nuclear localization signal (nls) LacZ reporter (RNZ) mouse and obtained the *Avil*-Cre:RNZ mouse. X-Gal staining in the intact TG of this mouse at a neonatal stage (P4–P6) revealed the distribution of sensory neurons along the dorsal surface of the TG (Fig. 1b). The newly developed *Avil*-nlsRFP transgenic mouse (PB, TS, TI: unpublished) that expresses the red fluorescent protein (RFP) in the nuclei of peripheral sensory neurons also demonstrated the sensory neuron distributions in the TG (Fig. 1c).

Based on the innervation target of peripheral processes, the TG has three major branches: the ophthalmic branch (V1) that innervates the eye, the maxillary branch (V2) that innervates the whisker pad, and the mandibular branch (V3) that innervates the lower jaw. To identify the region of TG that houses neurons innervating the large whiskers, we placed DiI crystals around individual whisker follicles and observed the region of subsequent dye labeling. DiI applied around the A-row whisker follicles stained neurons whose cell bodies are on the anteromedial side of the dorsal surface of the TG (Fig. 1d), and DiI application around the E-row follicles stained neurons on the more lateral side (Fig. 1e). These results demonstrate that a whisker-row-dependent topography exists along the dorsal surface of TG. On the other hand, when we placed DiI crystals around the follicles of C1 and C6 whiskers of different mice, no obvious difference in the localization of the labelled neurons was observed in the TG (Supplementary Fig. 1), which is consistent with a previous work suggesting that for the neurons innervating separate whiskers in the same row, the degree of their cell body segregation in TG is lower than that for neurons innervating whiskers in different rows^35^. We also placed DiI crystals in the lower jaw and observed subsequent labeling on the posterolateral side of the TG, implying that neurons in that region innervate the lower jaw (Fig. 1f), which is consistent with previous reports^36–38^. Hence, there exists an obvious topography in neuronal localization in the dorsal surface of TG depending on the projection sites of peripheral processes. Therefore, we identified the region of an intact TG that has neurons innervating the follicles of the large whiskers (Fig. 1g).

To test our hypothesis that the whisker-innervating region of TG generates spontaneous activity during neonatal stages, it is crucial to monitor neuronal activity in the intact TG, which was transected from other parts of the ascending trigeminal pathway. Thus, we established an ex vivo imaging system for neonatal TG. We crossed the *Avil*-Cre mouse with the *R26*-LSL-GCaMP6s reporter (*R26*-GCaMP6s) mouse to express the genetically encoded calcium indicator GCaMP6s in TG neurons (Fig. 1h). The TG is a fragile organ, so we utilized the whole TG, still attached to the cranial base for ex vivo imaging, by simply transecting its peripheral and central branch points to limit damage to the organ and retain its cytoarchitecture and microenvironment (Fig. 1i).

### Spontaneous activity is observed in neurons of the whisker-innervating region in neonatal TG

**Figure 2:**
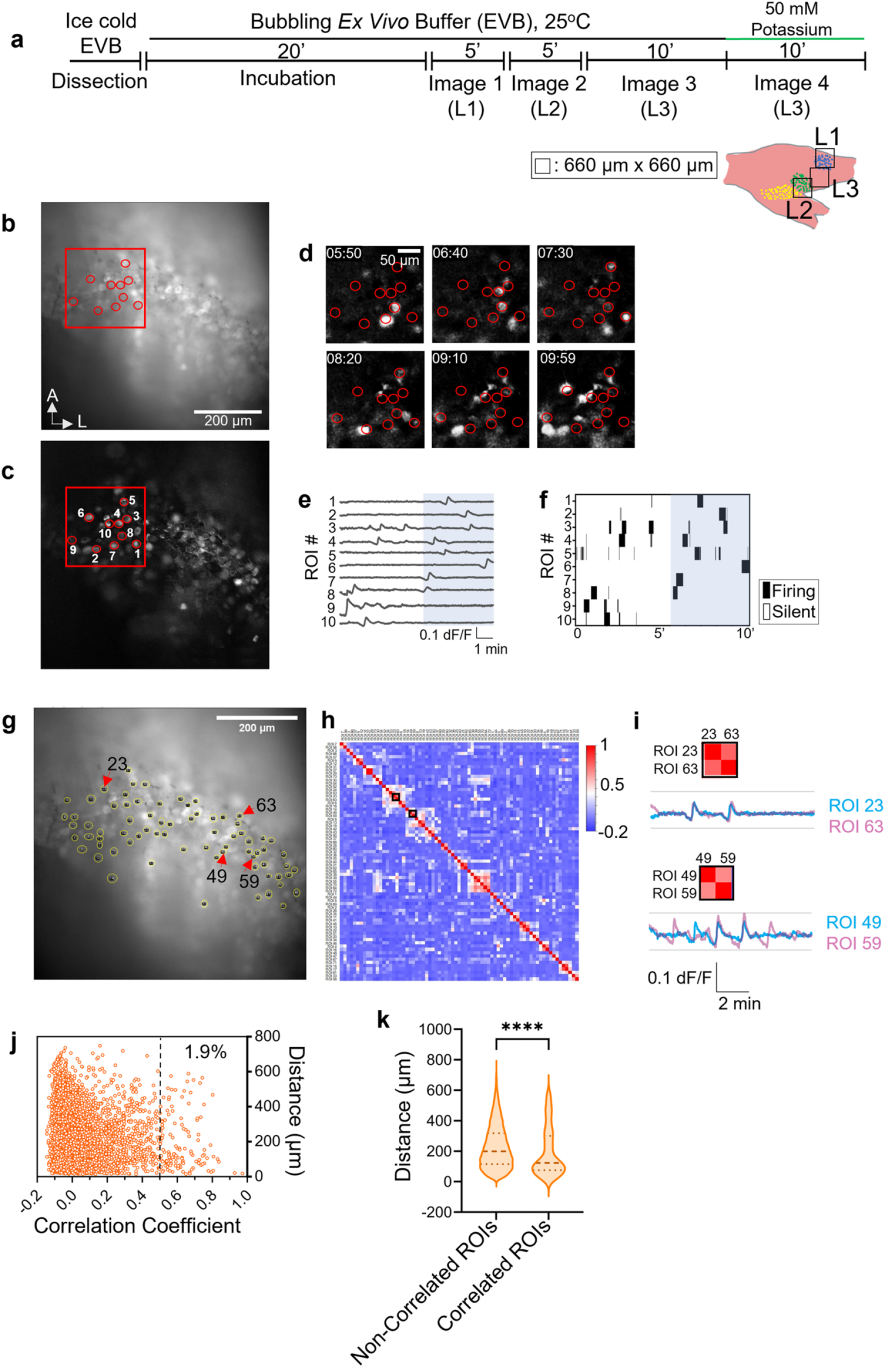
Spontaneous activity observed in whisker-innervating region of neonatal TG. (**a**) Experimental schematic for performing calcium imaging in a mouse TG ex vivo. See “Methods” for details. (**b**) A representative raw image frame of ex vivo calcium imaging from a total of 600 frames recorded over a period of 10 min (Image 3). The red circles indicate ten exemplary regions of interest (ROIs) of spontaneously firing neurons. (**c**) Standard deviation image of all 600 frames recorded as indicated in (**b**). The red circles indicate the same ROIs labelled in (**b**). **d**) Background subtracted (f − fo) images of the red square shown in (**b**) and (**c**) at six different timepoints taken at 50 s interval (350th–599th frame). (**e**) Calcium transients of the ten example ROIs in the 10 min duration. The blue rectangle indicates the range of the timepoints shown in (**d**) (350th–599th frame). (**f**) Binary raster plot for the ten example ROIs. Only the calcium transients exceeding the threshold are counted as firing. (Firing threshold: Mean + Std. Dev.). (**g**) Same image as (**b**) revealing the ROIs of all the detected spontaneously firing neurons in the 10 min duration. The yellow circles mark individual ROIs. (Total: 73 ROIs). (**h**) Representative correlation matrix calculated from the fluorescent signals of all the ROI pairs shown in (**g**) (Firing threshold: Mean + Std Dev). The black squares outline exemplary ROI pairs 23–63 and 49–59, respectively. (**i**) Fluorescence signals of two example ROI pairs having high correlation (> 0.5). The pair shown above (ROIs 23 and 63) are located far (339.3 µm) from each other and the pair shown below (ROIs 49 and 59) are located close (76.4 µm) to each other (see (**g**)). Correlation matrix of these exemplary ROIs is shown. (**j**) The plot compares the Pearson’s correlation coefficients of all ROI pairs with the distance between them. Each circle represents an individual ROI pair. The dashed vertical line separates the non-correlated ROI pairs (correlation coefficient ≤ 0.5) from correlated ones. (N = 4 animals). (**k**) Violin plot showing the distribution of all the correlated and non-correlated ROI pairs based on their inter-ROI distance. The dashed horizontal line represents the median, and the two dotted lines represent the quartiles. Two-tailed Mann–Whitney test was performed to check for significance (*p* value < 0.0001) (Total non-correlated ROI pairs = 6547, Total correlated ROI pairs = 129, N = 4 animals).

Using our ex vivo setup (Fig. 1i), we performed calcium imaging in the whisker-innervating region of TG isolated from the *Avil*-Cre:*R26*-GCaMP6s mice at P4–P6. In our experimental design, to cover the entire whisker-innervating area of TG, three regions were imaged: locus 1 (L1), locus 2 (L2), and locus 3 (L3), following which the organ was perfused with high potassium buffer to confirm its viability (Fig. 2a). We detected clear spontaneous activity in all of the L1, L2, and L3 regions at a cellular resolution (Fig. 2b–d; Supplementary Movie 1). Most neurons fired infrequently and did not exhibit obvious oscillatory calcium transients (Fig. 2e,f). At the population level, the spontaneous activity exhibited no recognizable synchronization for most of the active neurons, thereby appearing random, both spatially and temporally. Thus, we detected spontaneous activity in neurons of neonatal TG’s whisker-innervating region. Since this spontaneous activity occurred independent of whisker input, it originated within the TG itself.

To examine more systematically if spontaneous activity occurring in the neonatal TG was random or correlated, we placed regions of interest (ROIs) over all neurons that fired during the 10 min imaging session (Image 3) (Fig. 2g). Next, we calculated the Pearson’s correlation coefficient for fluorescent signal intensity between all the possible ROI pairs, which we then visualized with a color-coded correlation matrix (Fig. 2h,i). No obvious cluster was identified, and only ~ 1.9% of ROI pairs showed correlated firing, where ROI pairs having a correlation coefficient > 0.5 were considered to be correlated. To evaluate if the correlated pairs of neurons are located close to one another, we calculated the distance between all the possible ROI pairs and compared it with their respective correlation coefficients (Fig. 2j). We found that the distance between correlated ROI pairs (median: 123 µm) was significantly smaller than the distance between non-correlated ROI pairs (median: 199 µm), suggesting that correlated ROI pairs are majorly located closer to one another when compared with non-correlated ones (Fig. 2k). Overall, it appears that the spontaneous activity in neonatal TG is spatiotemporally sparse, with a small number of neuron pairs exhibiting some evident correlation.

### Spontaneous activity in TG is a characteristic of the early postnatal period

**Figure 3:**
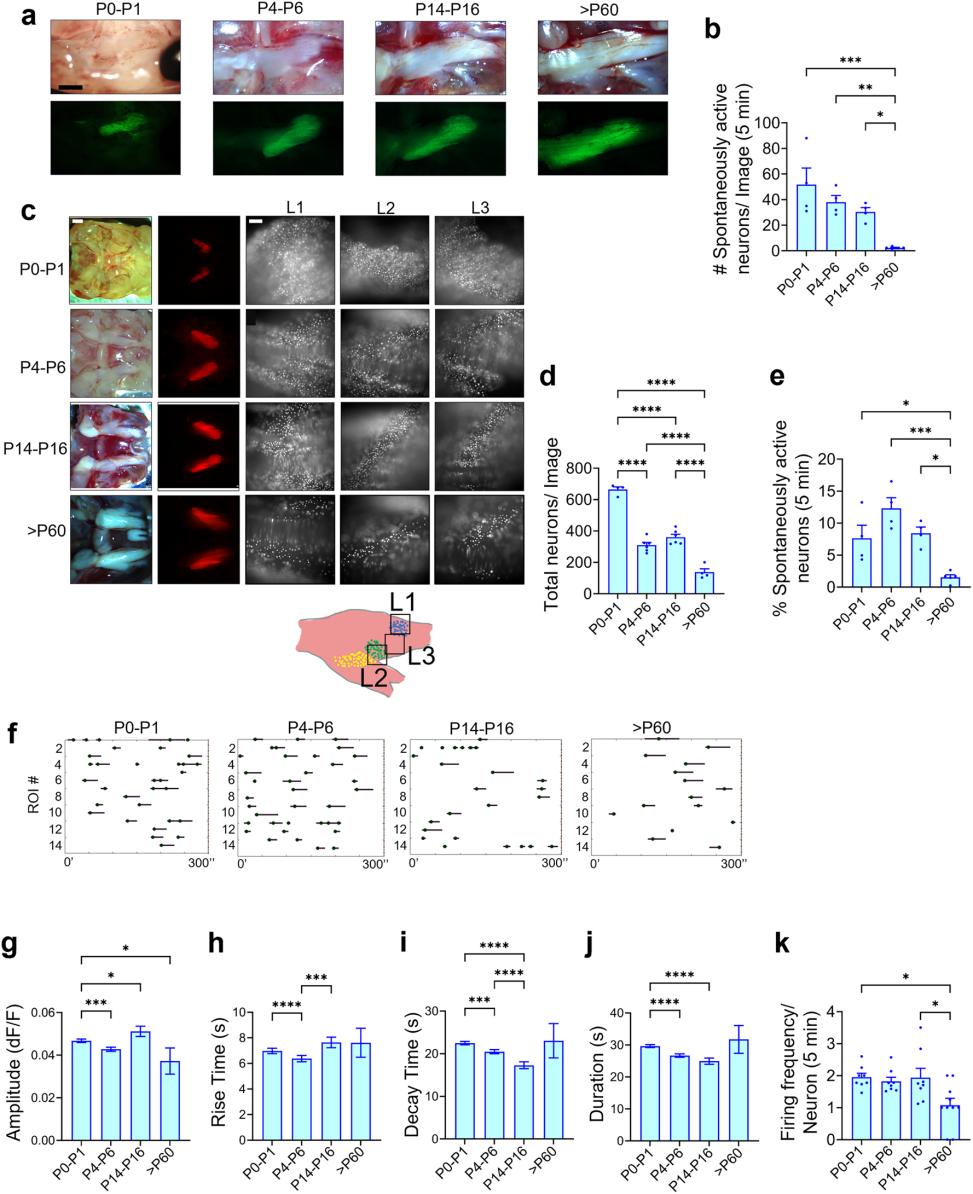
Spontaneous activity in TG diminishes by adult stage. (**a**) Representative images of *Avil*-Cre:*R26*-GCaMP6s mouse TG across four stages of development (P0–P1, P4–P6, P14–P16, > P60). Top panels show the brightfield images and the bottom panels show the GCaMP6s images. (Scale: 1 mm). (**b**) Bar graph showing the number of spontaneously firing neurons in a duration of 5 min (Avg. of Images 1, 2, and 3) across the four stages of development. One-way ANOVA with Tukey’s multiple comparison test is performed. Each dot represents an individual animal. [P0–P1 (n = 4 animals), P4–P6 (n = 4), P14–P16 (n = 4), > P60 (n = 5)]. (**c**) The first column shows the brightfield image of *Avil*-nlsRFP mouse TG across the four stages of development. The second column shows the RFP + area of *Avil*-nlsRFP mouse TGs. The last three columns show regions (L1, L2, L3) of the *Avil*-nlsRFP mouse TG in the ex vivo setup. (Scales: 1 mm for first two columns and 100 µm for last three columns). (**d**) Bar graph representing the number of nuclei (neurons) visible in one locus of ex vivo imaging across the four stages of development (Avg. of L1, L2, and L3). One-way ANOVA with Tukey’s multiple comparison test is performed. [P0–P1 (n = 4 animals), P4–P6 (n = 6), P14–P16 (n = 6), > P60 (n = 4)]. (**e**) Normalized data of the percentage of neurons firing spontaneously across the four stages of development. In adult stage, < 2% neurons fire spontaneously, which is significantly less compared to the remaining three stages. One-way ANOVA with Tukey’s multiple comparison test is performed. (**f**) Raster plots showing spontaneous activity status of 14 randomly selected ROIs in a duration of 5 min at each of the four stages of development. The circle signifies the position of the peak, the line before the circle shows the rise time, and the line following the circle shows the decay time of individual calcium transients. (Firing Threshold: Mean + Std. Dev.) [P0–P1 (n = 4 animals), P4–P6 (n = 4), P14–P16 (n = 4), > P60 (n = 5)]. (**g**) Comparison of average amplitude of calcium transients across the four stages of development. Kruskal–Wallis test is performed to check for significance. [P0–P1 (n = 870 peaks, 4 animals), P4–P6 (611 peaks, n = 4), P14–P16 (322 peaks, n = 4), > P60 (29 peaks, n = 5)]. (**h**) Comparison of average rise time of calcium transients across the four stages of development. Kruskal–Wallis test is performed to check for significance. [P0–P1 (870 peaks, n = 4 animals), P4–P6 (611 peaks, n = 4), P14–P16 (322 peaks, n = 4), > P60 (29 peaks, n = 5)]. (**i**) Comparison of average decay time of calcium transients across the four stages of development. Kruskal–Wallis test is performed to check for significance. [P0–P1 (702 peaks, n = 4), P4–P6 (501 peaks, n = 4), P14–P16 (258 peaks, n = 4), > P60 (15 peaks, n = 5)]. (**j**) Comparison of average duration of calcium transients across the four stages of development. Kruskal–Wallis test is performed to check for significance. [P0–P1 (702 peaks, n = 4), P4–P6 (501 peaks, n = 4), P14–P16 (258 peaks, n = 4), > P60 (15 peaks, n = 5)]. (**k**) Comparison of average firing events for individual neurons in a duration of 5 min across the four stages of development. One-way ANOVA with Tukey’s multiple comparison test is performed. [P0–P1 (n = 359 ROIs, 4 animals), P4–P6 (n = 326 ROIs, 4 animals), P14–P16 (n = 218 ROIs, 4 animals), > P60 (n = 23 ROIs, 4 animals)]. (All error bars: SEM).

To compare spontaneous activity in TG across different stages of development, we considered four age groups: P0–P1 (newborn stage), P4–P6 (neonatal stage), P14–P16 (juvenile stage), and > P60 (adult stage), and performed ex vivo calcium imaging using *Avil*-Cre:*R26*-GCaMP6s mice (Figs. 2a and 3a). The newborn stage was selected to elucidate if spontaneous activity in TG initiates before the formation of the barrel map; the juvenile stage was selected to determine if spontaneous activity continues in the second postnatal week, and the adult stage was selected to reveal whether or not TG spontaneous activity is specifically a characteristic of the early-postnatal period. At P0–P1, the TG exhibited clear spontaneous activity with ~ 50 neurons firing spontaneously in an imaging locus (660 × 660 µm) in a 5 min duration (Avg. of Images 1, 2, and 3), which was similar to the P4–P6 stage wherein ~ 40 neurons fired spontaneously in the same duration (Fig. 3b; Supplementary Movie 2). We also observed spontaneous activity in the P14–P16 stage with ~ 30 neurons firing spontaneously in a 5 min duration (Fig. 3b; Supplementary Movie 3). Similar to the P4–P6 stage, in the P0–P1 and P14–P16 stages, no obvious pattern or correlated firing of a large group of neurons was identified, nor did the neurons seem to exhibit an oscillatory firing pattern. Contrary to the first 2 postnatal weeks, minimal to no spontaneous activity was observed in adult TG, with < 5 neurons firing spontaneously in a 5 min duration (Fig. 3b; Supplementary Movie 4). We evaluated the viability of the TG by subjecting it to high potassium buffer (Image 4) after EVB (Fig. 2a) and found that TG neurons in all stages reacted to high potassium irrespective of whether or not they fired spontaneously (Supplementary Fig. 2), thereby confirming that the explant was viable and healthy. Consequently, despite the adult TG’s viability, spontaneous activity was greatly reduced, indicating that it was a hallmark of that stage.

The TG evidently increased in size during development, as did the GCaMP^+^ area (Fig. 3a). Therefore, the total number of neurons in each imaging locus was likely to decline with age. To quantify this, we used the *Avil*-nlsRFP mouse. First, we calculated the area of the nlsRFP^+^ region across the ages to see the factor by which it increases with development and found that it almost quadruples by the adult stage when compared with the P0–P1 stage (Fig. 3c; Supplementary Fig. 3). Next, we quantified the total number of nuclei (neurons) in L1, L2, and L3 across the stages of development (Fig. 3c). Expectedly, the number of neurons in each locus significantly decreased with age, from > 600 neurons/image in the P0–P1 stage, to 300–350 neurons/image in the P4–P6 and P14–P16 stages, and < 200 neurons/image in the adult stage (Avg. of L1, L2, and L3) (Fig. 3d). We calculated the percentage of neurons firing spontaneously across the stages of development by dividing the number of spontaneously firing neurons (Fig. 3b) by the total number of neurons visible in each imaging region (Fig. 3d). Our results revealed that in the P4–P6 stage, ~ 12% of neurons fire spontaneously, whereas in the adult stage, < 2% of neurons fire spontaneously (Fig. 3e), thereby confirming that spontaneous activity in TG neurons significantly diminishes by the adult stage.

Additionally, we compared the firing characteristics of spontaneous activity in TG neurons across development (Fig. 3f). The amplitude of calcium transients significantly fluctuated across development (Fig. 3g), as did the rise time of individual calcium transients across the first 2 postnatal weeks (Fig. 3h). The decay time and total duration of individual calcium transients decreased across the first 2 postnatal weeks (Fig. 3i,j). Furthermore, we found that by the adult stage, along with the decline in the total number of spontaneously firing neurons, their frequency of firing also decreases when compared with the first 2 postnatal weeks (Fig. 3f,k). It should be noted that these firing parameters may not be very accurate since our priority was to detect spontaneous activity in the TG, and therefore, we used GCaMP6s, which is more sensitive but has slower kinetics than GCaMP6f.^39^, as a calcium indicator. Also, it is unclear whether the firing characteristics differences detected between the neonatal period and adulthood are ascribed to the developmental changes of firing properties of the same neuronal population. Instead, it is likely that the populations of firing neurons themselves are different between these two ages, because spontaneously active neurons were much less in adulthood than in neonates (Fig. 3e).

To conclude, we found the existence of spontaneous activity in TG across the first 2 postnatal weeks, with subtle changes in their firing patterns across development, implying that spontaneous activity is evolving with age.

### Majority of spontaneously firing neurons in neonatal TG has medium-to-large diameter

**Figure 4:**
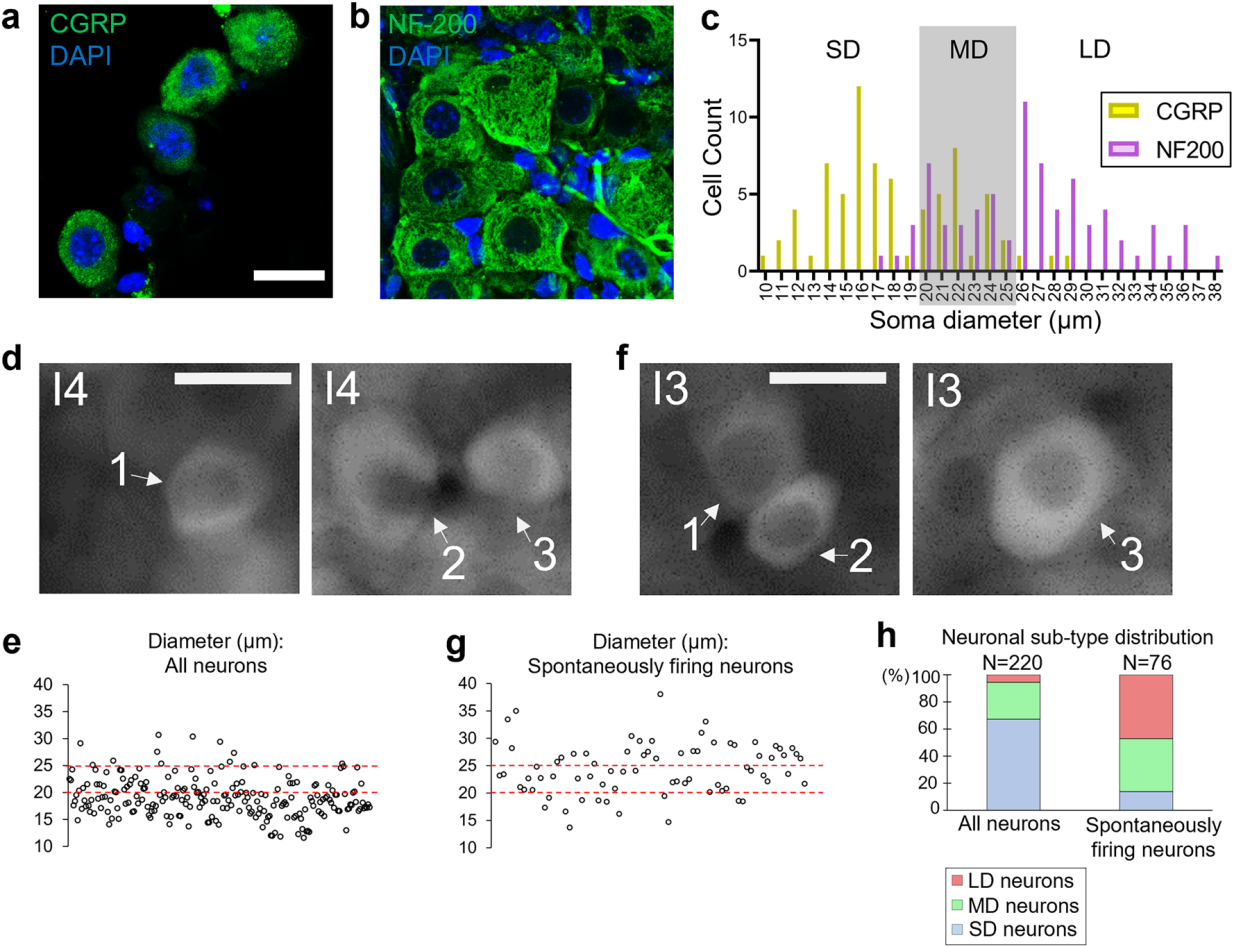
Spontaneous activity in neonatal TG is majorly exhibited by medium-to-large diameter neurons. (**a**, **b**) Representative images showing CGRP^+^ (**a**) and NF-200^+^ (**b**) neurons in 20 µm-thick longitudinal sections of mouse TG at P6. Nuclei were stained by DAPI. (**c**) Distribution of soma-diameter of CGRP^+^ and NF-200^+^ neurons (CGRP^+^ : n = 74 neurons, NF-200^+^ : n = 75 neurons, 2 animals each). SD, MD, and LD: small, medium, and large-diameter neurons, respectively. (**d**) Stack images as the sum of all 600 frames recorded over a 10 min duration of ex vivo imaging when neurons were subjected to high potassium buffer (Image 4 (I4), L3) for three exemplary ROIs. Diameter of ROIs 1, 2, and 3 is 18.6 µm, 25.5 µm, 18.4 µm, respectively. (**e**) Scatter plot showing the distribution of neurons activated by high potassium by the diameter of their soma. The lower dashed line represents the boundary of SD and MD neurons (20 µm). The upper dashed line represents the boundary of MD and LD neurons (25 µm). (n = 220 neurons, 2 animals). (**f**) Stack images as the sum of all 600 frames recorded over a 10 min duration of ex vivo imaging when neurons were perfused with EVB (Image 3 (I3), L3) for three exemplary ROIs. Diameters of ROIs 1, 2, and 3 are18.2 µm, 19.0 µm, 26.0 µm, respectively. (**g**) Scatter plot showing the distribution of spontaneously firing neurons by the diameter of their soma. (n = 76, neurons, 2 animals). (**h**) Stacked bar graph comparing the distribution of all neurons and spontaneously firing neurons based on their soma-diameter. The total number of neurons considered is indicated above the bars. Scale bars: 20 µm.

The rodent TG has three major sensory neuronal subtypes based on the diameter of the soma: small diameter (SD) is a characteristic of nociceptive neurons, and medium diameter (MD) to large diameter (LD) are characteristics of mechanosensory neurons. Previous studies in peripheral sensory neurons of adult mice claimed that the diameter of SD, MD and LD neurons is < 20 µm, 20–25 µm, and > 25 µm, respectively^40^. To estimate the size of sensory neuron subtypes in neonatal TG, we used calcitonin gene-related peptide (CGRP) and neurofilament-200 (NF-200) as phenotypic markers to differentiate between SD-MD, and MD-LD neurons, respectively, and performed immunostaining in P6 TG sections (Fig. 4a,b). Our results showed that the diameter of CGRP^+^ neurons and NF-200^+^ neurons are 18.11 ± 4.26 µm and 26.36 ± 4.88 µm, respectively. Our data demonstrating the distribution of neurons in neonatal TG based on their soma-diameter was similar to that shown in adult peripheral sensory neurons; thus, we used the same definition of SD (< 20 µm), MD (20–25 µm), and LD (> 25 µm) neurons as reported previously (Fig. 4c)^40^.

We then quantified the total population of neurons in P4–P6 TG based on their soma size. To activate the majority of the sensory neurons, we subjected the *Avil*-Cre:*R26*-GCaMP6s mouse TG to high potassium buffer ex vivo (Image4 (I4)) (Fig. 2a). Then, we measured the diameter of each neuron in an imaging locus (L3) in which the soma boundary was clearly visible (Fig. 4d). We found that 67.3%, 27.3%, and 5.5% of neurons are SD, MD and LD neurons, respectively (Fig. 4e,h) (n = 220 neurons, 2 animals). This was similar to previous reports in adult rat TG which suggest that > 90% of neurons are SD-MD neurons^41–43^. Next, we chose neurons that fired spontaneously in a 10 min duration (Image3 (I3)) (See Fig. 2a) and whose soma-boundary was clearly visible to measure their respective diameters (Fig. 4f). We found that 17.1%, 39.5%, and 43.4% were SD, MD, and LD neurons, respectively (Fig. 4h). It is important to note that 82.9% of spontaneously firing neurons were MD-LD neurons, the majority of which are likely to be mechanosensory neurons (Fig. 4h) (n = 76 neurons, two animals).

Since ~ 12.3% of P4–P6 neurons fired spontaneously in a duration of 5 min (Fig. 3e), we calculated the active proportion in each neuronal subtype population and roughly estimated that 3% (≒ 12.3 × 17.1 / 67.3), 18% (≒ 12.3 × 39.5 / 27.3), and 98% (≒ 12.3 × 43.4 / 5.5) of SD, MD, and LD neurons, respectively, fired spontaneously in a duration of 5 min. Hence, the majority of LD neurons and a negligible fraction of SD neurons fire spontaneously in neonatal TG ex vivo.

### Chelation of extracellular calcium blocks spontaneous activity in neonatal TG

**Figure 5:**
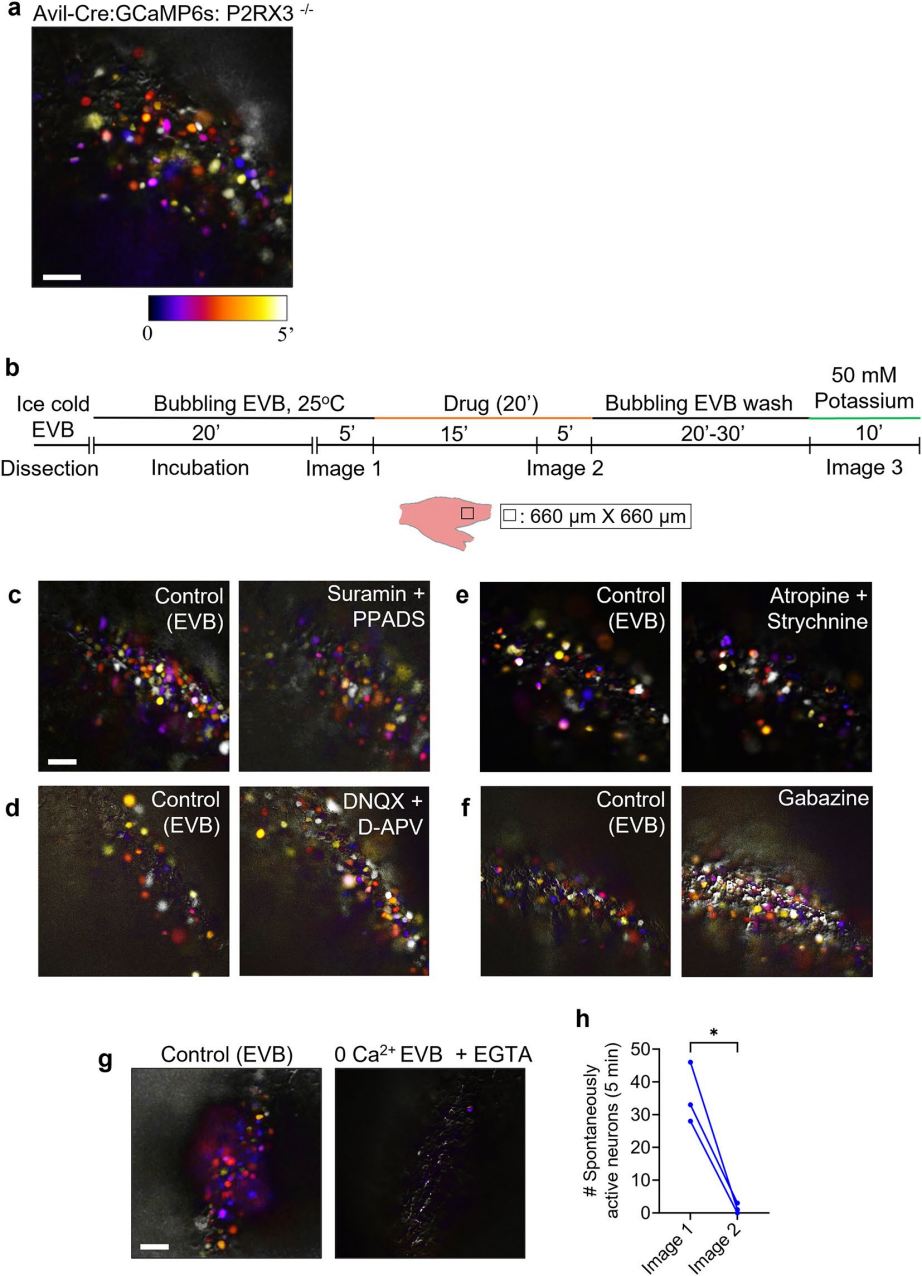
Chelation of extracellular calcium blocks spontaneous activity in neonatal TG. (**a**) Representative temporal color-coding map showing all spontaneously active neurons in the TG of an *Avil*-Cre:*R26*-GCaMP6s:*P2RX3*^−/−^ mouse (age: P5) in a duration of 5 min (n = 3). (**b**) Experimental schematic for testing drugs in ex vivo calcium imaging of P4–P6 TG. See the “Methods” for details. (**c**–**f**) Temporal color-coding maps showing all spontaneously active neurons in a duration of 5 min (Image 1 (I1)-Left, Image 2 (I2)-Right). (**c**) Broad purinergic receptor blockers: Suramin (500 µM) + PPADS (100 µM) did not have any obvious effect on spontaneous activity in TG. (**d**) Broad AMPA/Kainate/NMDA receptor blockers: DNQX (100 µM) + D-APV (50 µM) did not have any obvious effect on spontaneous activity in TG. (**e**) Broad cholinergic/glycinergic receptor blockers: Atropine (15 µM) + Strychnine (5 µM) did not have any obvious effect on spontaneous activity in TG. (**f**) Broad GABA-A receptor blocker: Gabazine (100 µM) did not have any obvious effect on spontaneous activity in TG. (**g**) Representative temporal color-coding map showing the number of spontaneously active neurons in a duration of 5 min before (Image 1) and after (Image 2) perfusion with 0 Ca^2^^+^ EVB + EGTA (50 mM). (**h**) Quantitative analysis of the change in the number of spontaneously active neurons following chelation of extracellular calcium (Paired two-tailed t test, p value: 0.0236, N = 3 animals). Scale bars: 100 µm.

To elucidate the mechanism generating spontaneous activity in P4–P6 TG, we primarily focused on purinergic receptors as potential candidates since these molecules are known to play a key role in the generation of spontaneous activity in developing mouse cochlea^25,44^. Our RNA-seq data for P5 TG revealed that *P2RX3* gene has the highest expression among all the purinergic receptor genes expressed (Supplementary Fig. 4a; Supplementary Table 1). Our quantitative reverse transcription-PCR (qRT-PCR) confirmed that *P2RX3* gene is highly expressed in the TG but not in the brain at P5 (Supplementary Fig. 4b). To test if *P2RX3* plays a role in generating spontaneous activity in neonatal TG, we generated *P2RX3* knockout mice using CRISPR/Cas9 (Supplementary Fig. 5). Then we performed ex vivo calcium imaging in P4–P6 TG of *P2RX3* knockout mice that express GCaMP6s in the TG neurons (*Avil*-Cre: *R26*-GCaMP6s: *P2RX3*^−/−^ mice). However, contrary to our expectation, we observed clear spontaneous activity (n = 3) (Fig. 5a). Parallelly, to widen our scope from *P2RX3* to all the purinergic receptors expressed, we tested their broad pharmacological inhibitors (Suramin and PPADS) for the TG prepared from wild-type mice by using our ex vivo imaging system (Fig. 5b). These too did not have any obvious effect on perturbing spontaneous activity in neonatal TG (Fig. 5c). These results indicate that *P2RX3* is not essential for the generation of spontaneous activity in the neonatal TG. We next tested the broad inhibitors of ionotropic glutamate receptors (DNQX + APV), cholinergic receptors (Atropine), glycinergic receptors (Strychnine), and GABA receptors (Gabazine) in TG ex vivo since each of these is known to be involved in the generation of spontaneous activity in other developing systems such as retinal ganglion cells, spinal cord, hippocampus, etc.^6,17,45–49^. Nevertheless, none of these perturbed or inhibited the spontaneous activity in neonatal TG (Fig. 5d–f). Thus, these neurotransmitter receptors had no obvious effect on spontaneous activity in neonatal TG.

Lastly, we wanted to delineate the role of extracellular calcium in generating spontaneous activity in the TG. We tested the calcium chelator (EGTA) in calcium-free EVB (0 Ca^2+^ EVB), and found that it almost completely blocked spontaneous activity in P4–P6 TG of *Avil*-Cre:*R26*-GCaMP6s mice (n = 3) (Fig. 5g,h). After washing it out, the organ reacted robustly to high potassium buffer, indicating that the TG was still viable (Supplementary Movie 5). This result indicates that extracellular calcium plays a crucial role in generating spontaneous activity in neonatal TG.

## Discussion

The present study provides the first evidence of the occurrence of spontaneous activity in the peripheral structures of the somatosensory system during the early postnatal period. Spontaneous activity in the developing sensory systems is important for the refinement of neural circuits. By establishing a method of ex vivo calcium imaging, we discovered that neurons of the TG that transmit tactile sensation from the whiskers to the brainstem exhibit spontaneous activity during the first postnatal week (Figs. 1 and 2). This activity is largely sporadic, with no obvious spatiotemporal pattern or wide-scale co-activation of neurons (Figs. 2 and 3). Even so, a small population of neuronal pairs, which tend to localize close to each other, shows some evident correlation. Spontaneous activity in the TG is also observed during the second postnatal week but majorly diminishes by adulthood (Fig. 3). Additionally, the majority of the spontaneously firing neurons have a medium-to-large diameter, which is a characteristic of mechanosensory neurons (Fig. 4). Furthermore, this activity is blocked by chelation of extracellular calcium and, in accordance with our pharmacological study data, it may originate in a cell-intrinsic manner (Fig. 5).

### Spontaneous activity in the somatosensory periphery during the neonatal stage

Spontaneous activity in the developing nervous system has been well studied in the peripheral structures of the visual and auditory systems. In the visual system, synchronous bursts are observed in the retinal ganglion cells of isolated neonatal retinas, which propagate tangentially across the organ in a wave-like manner^14–17^. Similarly, in the auditory system, spontaneous activity is observed in the spiral ganglion cells and inner hair cells from the isolated neonatal cochlea^20–25^. In the visual system, the spontaneous activity in the immature retina is transmitted to the visual cortex and superior colliculus, wherein, the wave-like pattern is also preserved^4,50^. Similarly, in the auditory system, the spontaneous activity in the developing cochlea is transmitted to the auditory cortex and inferior colliculus, wherein the activity is observed in accordance with the future tonotopic axis^18^. During the first postnatal week, neurons in barrel cortex L4 exhibit correlated spontaneous activity, where neurons within the same barrel tend to fire together, giving a patchwork-like appearance^5,7,8,26–30^. Along with L4 neurons, the thalamocortical axons also exhibit patchwork-type spontaneous activity in the barrel cortex L4 in neonates^5^.

Our current discovery of spontaneous activity in the whisker-innervating neurons of the neonatal TG (Figs. 1 and 2) underscores the likelihood of it being the source of spontaneous activity in the cortex. Since we used a TG transected from all upstream afferents and inputs, it clearly indicates that the spontaneous activity observed in TG neurons originates within the organ itself (Figs. 1 and 2). Furthermore, we found that more than 80% of the spontaneously firing neurons are medium-to-large-diameter neurons, which are likely the mechanosensory neurons (Fig. 4). Mechanosensory neurons play a pivotal role in transmitting and processing tactile information from individual whiskers to the barrel cortex. Thus, spontaneous activity in these neurons during the early postnatal period may aid in the refinement of the ascending somatosensory pathway which in turn leads to precise sensory processing in the later stages.

We further found that the spontaneous activity in the TG is a likely characteristic of the early postnatal period since it was majorly diminished by the adult stage, wherein only a small population (< 2%) of neurons fired spontaneously (Fig. 3). On the other hand, it was somewhat unexpected to observe spontaneous activity in the TG neurons during the second postnatal week (P14–P16), because the cortical spontaneous activity no longer arrives via thalamocortical axons by P9^7^. However, a similar trend is reported in the visual and auditory systems, wherein the peripheral structures continue to exhibit spontaneous activity even during the second postnatal week^25,45,51^. In the mouse visual system, the retinal spontaneous activity observed at P10–P12 is transmitted to the thalamus and superior colliculus but not to the cortex^52^. The interpretation of this phenomenon is that the retina-derived spontaneous activity could be masked by cortical activity when the visual cortex is being matured ^52^. Although the timing of cortical maturation may be slightly different among different sensory modalities, by the second postnatal week, majority of inputs even to the barrel cortex become cortico-cortical, as opposed to the majority of inputs being thalamo-cortical during the first postnatal week^53–57^. Hence, it is likely that although the TG continues to exhibit spontaneous activity by the P14–P16 stage, it is no longer a source of the cortical spontaneous activity by that stage.

The thalamus and whisker pads have also been candidates as probable sources for the spontaneous activity observed in the neonatal barrel cortex. In the thalamocortical slice prepared from prenatal mouse, wave-type spontaneous activity originates in the thalamus (Moreno-Juan et al. 2017; Anton-Balanos et al. 2019). Nonetheless, injection of a local anesthetic to the contralateral whisker pad in the neonatal mouse blocks the cortical spontaneous activity^5^. This result suggests that, at least in the neonatal stage, the source is most likely in the periphery itself, thereby excluding the brainstem and thalamus as possibilities. On the other hand, the TG has pseudo-unipolar sensory neurons whose peripheral afferents innervate the whisker pad, which explains how it can be silenced by a local anesthetic. Sensory feedback from inadvertent motor activity in the periphery, such as self-generated spontaneous whisker movements, are also suggested to induce spontaneous activity in the developing barrel cortex and thalamus before the onset of exploratory whisker movement^31–34^. However, in our previous study, the majority (89%) of patchwork-type activity in the neonatal barrel cortex L4 was not associated with corresponding whisker movements^5^. In addition, transection of the ION did not block the cortical L4 spontaneous activity^5^, suggesting that a major source is likely to be downstream of the ION. In the present study, by establishing a novel ex vivo imaging system, we demonstrated the presence of spontaneous activity in the neonatal TG, which is likely a major source of the spontaneous activity that is observed in the neonatal barrel cortex.

### How does the patchwork-type pattern of cortical spontaneous activity emerge?

If the TG is the source of cortical spontaneous activity, where and how does the patchwork-type pattern emerge? We did not identify any obvious pattern or wide-scale co-activation of a large group of neurons in the neonatal TG (Fig. 2). Still, we were able to find a small population of neuronal pairs, that tend to be located close to each other and exhibit some evident correlation. These neurons may possibly innervate the same whisker follicles. In the TG, there exists a weak whisker-dependent topography in neuronal soma localization. Although there is no obvious clustering for the group of neurons that innervate the same whisker, they tend to be located close to each other^35–38^. Therefore, it is possible that the neurons that innervate the same whisker follicle tend to fire together, and in this scenario, the TG should already have a template for the patchwork-type pattern that gets transmitted to the cortex via the brainstem and thalamus. Another possibility is that the spontaneous activation of a single TG neuron further activates multiple brainstem neurons within the same barrelette, which subsequently activates a single barreloid in the thalamus. Thus, the activity from one TG neuron may amplify at each relay station to ultimately activate multiple neurons within the same barrel, thereby forming a “patch”, and hence revealing the patchwork-like pattern observed in the cortex. In this scenario, the spontaneous activity originates from the TG, but the patchwork-type pattern shapes in the brainstem. A recent study by our group demonstrated that transection of ION at P0–P1 disrupts the pattern of cortical spontaneous activity observed at P5^58^. Therefore, in either of the scenarios discussed above, the template of the patchwork-type pattern should be generated depending on the inputs from the whiskers in the newborn stage, such as P0–P1.

### Mechanism generating spontaneous activity in TG

In the developing rodent retina, spontaneous activity has three sequential stages, each with its own mechanism: stage I waves (E16-P0) are inhibited by blockers of gap junctions and adenosine receptors, stage II waves (P0–P9) are inhibited by blockers of acetylcholine and GABA receptors, and stage III waves (P10–P14) are inhibited by blockers of glutamate and muscarinic receptors^47,59–61^. In the developing cochlea, spontaneous activity is inhibited by blockers of purinergic receptors^25,62^. Unlike the retina and cochlea, the isolated TG does not have synapses. Hence, it is unlikely for neurotransmitters to play a role in generating spontaneous activity. This is validated by our findings in this study demonstrating that the spontaneous activity in TG is not inhibited/perturbed by pharmacological blockers of glutamatergic receptors, cholinergic receptors, GABA-A receptors, and glycinergic receptors, and purinergic receptors (Fig. 5). We assume that the spontaneous activity in TG might be generated in a cell-intrinsic manner. The surge in intracellular Ca^2+^ during spontaneous Ca^2+^ transients is either due to the influx of extracellular Ca^2+^, or due to release of Ca^2+^ from intracellular stores. We found that chelation of extracellular Ca^2+^ with EGTA blocked the TG spontaneous activity (Fig. 5). On the same note, a previous electrophysiological study demonstrated that induced action potentials in neonatal TG neurons were highly sensitive to reductions in extracellular calcium in vitro^63^. Thus, extracellular Ca^2+^ plays a crucial role in the generation of neonatal TG spontaneous activity (Fig. 5). Among the 5 given sub-classes of calcium channels (HGNC classification), only the voltage-gated calcium channels (VGCCs) can cause the extracellular calcium to affect the spontaneous activation of the cells (Supplementary Table 2). Thus, it is likely that the influx of extracellular calcium via the VGCCs activated by spontaneous fluctuation in membrane potential generates spontaneous activity in P4–P6 TG.

In summary, we discovered spontaneous activity in the sensory neurons located in the whisker-innervating region of neonatal TG ex vivo, which is the first evidence of spontaneous activity in the peripheral structure of the developing somatosensory system. Our findings will help further our understanding of the role of spontaneous activity in the development and establishment of precise and mature sensory circuits.

## Materials and methods

### Animals

All experiments were performed according to the guidelines for animal experimentation of the National Institute of Genetics (NIG) and were approved by the animal experimentation committee of the NIG. Our study is in accordance with the ARRIVE guidelines. Sex of pups was not identified. PCR primers used for genotyping of *Avil*-Cre (RBRC10246)^35,64^, *R26*-LSL-nls-lacZ (RNZ) (RBRC02657)^65^, *R26*-LSL-GCaMP6s (*R26*-GCaMP6s) (Ai96: 024106)^66^, and *Avil*-nlsRFP (PB, TS, TI: unpublished) mice were KS149/KS150, KS109/KS110 and KS149/KS150, respectively (Supplementary Table 3).

### Generation of P2RX3 knockout mouse

*P2RX3* global knockout mice were generated using the CRISPR/Cas9 system^67,68^. To delete the putative promoter, transcriptional initiation site and translational initiation site of the *P2RX3* gene, single guide RNAs (sgRNAs) were designed flanking exon 1 of the *P2RX3* gene using the CRISPRdirect^69^ and CRISPOR^70^ tools. sgRNAs were selected based on high MIT and CFD score (> 90), low off-target mismatches, and their location with respect to exon 1. Based on these criteria, two sgRNAs were selected (sgRNA_62Forw, sgRNA_1228Forw) (Supplementary Table 3). Alt-R CRISPR-Cas9 crRNAs and Alt-R CRISPR-Cas9 tracrRNA were ordered from IDT (Integrated DNA Technologies). The annealed scRNAs and tracrRNA, and TrueCut Cas9 protein v2 (Invitrogen) were premixed and electroporated into fertilized eggs of B6/C3H F2 using CUY21EDIT II electroporator and LF501PT1-10 platinum plate electrode (BEX Co. Ltd.). Founder mice were screened by genotyping using primers KS253/254 and OP31/OP32 (Supplementary Table 3). Sequences of the four founder mice were determined to assess the deletion.

RT-PCR was used to confirm the absence of *P2RX3* transcripts in *P2RX3*^−/−^ (KO) mouse. TGs were carefully isolated from P4–P6 *P2RX3*^−/−^ (KO) and *P2RX3*^+/+^ (WT) littermates on ice, and immediately transferred to ice-cold RNALater solution (Sigma) in which they were incubated at room temperature for up to 1 week. RNA was isolated using the RNEasy Mini Kit (Qiagen, #74104) and stored in − 80 °C. cDNA was synthesized using the PrimeScript RT reagent kit (Takara, #RR037Q), and was used as templates for RT-PCR. Primer pairs OP11/OP12 and OP19/OP20 were used for *GAPDH* and *P2RX3*, respectively. The sequence for all the primers is mentioned in Supplementary Table 3.

### X-Gal staining

Male *Avil*-Cre mice were mated with female RNZ mice to obtain *Avil*-Cre:RNZ mice. *Avil*-Cre:RNZ pups were sampled at P4–P6, and the TG was isolated while still attached to the cranial base by specifically transecting its peripheral and central nerve terminals. Post-dissection, the whole TG was fixed in 4% paraformaldehyde (PFA) in 0.1 M phosphate buffer (PB) for 2 h at room temperature, and rinsed thrice with 0.05 M PB. The TG was stained overnight at 37 °C with X-Gal staining solution (5 mM potassium ferricyanide, 5 mM potassium ferrocyanide, 2 mM magnesium chloride, 1 mg/ml X-Gal in 0.05 M PB). The following day, the TG was rinsed thrice with 0.05 M PB and post-fixed in 4%PFA in 0.1 M PB overnight. Images were acquired using the M205 FCA microscope (Leica) and DFC7000T camera (Leica).

### DiI labelling

DiI crystals (Invitrogen, D282) were placed around individual whisker follicles of P0–P1 mice using a 35G beveled needle (World Precision Instruments, NF35BV-2), following which, the pups were warmed on a heating pad (37 °C) for 10–15 min before returning to the mother. Four to 5 days later, the pups were sampled, and the TG was carefully isolated while still attached to the cranial base. DiI labelling was observed in the TG and images were acquired using the M205 FCA microscope (Leica) and DFC7000T camera (Leica).

### Immunohistochemistry

At P6, pups (ICR) were anesthetized with pentobarbital intraperitoneal injection and perfused with 0.9% NaCl followed by 4% paraformaldehyde (PFA) in PB for fixation. The TG was isolated while still attached to the cranial base and post-fixed overnight in 4% PFA in 0.1 M PB at 4 °C. The following day, isolated TGs were washed with 0.1 M PB and transferred to 30% sucrose in 0.1 MPB at 4 °C till sectioning. For sectioning, TGs were carefully separated from the cranial base and fixed in OCT compound. 20 µm-thick longitudinal sections were made using a cryostat (Leica CM3050S) and mounted on MAS-coated slides (Matsunami, MAS-02). The slides were dried overnight at RT and immunostaining was performed. Slides were incubated in blocking buffer (3% goat serum, 0.5% TritonX-100 in phosphate buffer saline (PBS)) for 2 h at RT, following which, primary antibody solution was applied: CGRP (1:1000, Immunostar #24112), NF-200 (1:500, Sigma #N4142). The slides were incubated overnight on a shaker at 4 °C. The following day, the slides were washed with 1X PBS and incubated in secondary antibody solution: Alexa 488 (Goat anti-rabbit IgG, Invitrogen #A11034) for 1 h on a shaker at RT. After this, the slides were washed with 1X PBS and incubated in DAPI solution (1 ug/ml) for 2 min at RT. The slides were again washed with 1X PBS and mounting medium was applied followed by coverslip. The slides were incubated at 4 °C overnight and sealed the following day. Images were taken using a confocal microscope (Leica TCS SP5 II) and a 40X lens. Diameter of individual cells were calculated using Fiji ImageJ.

### Ex vivo calcium imaging of TG

The male *Avil*-Cre mice were mated with the female *R26*-GCaMP6s mouse to obtain *Avil*-Cre:*R26*-GCaMP6s pups. Ex vivo buffer (EVB) (pH 7.4) contained 135 mM NaCl, 5 mM KCl, 1.5 mM CaCl_2_, 1.5 mM MgCl_2_, 20 mM HEPES (pH 7.4) and 10 mM Glucose. High potassium buffer (pH 7.4) contained 90 mM NaCl, 50 mM KCl, 1.5 mM CaCl_2_, 1.5 mM MgCl_2_, 20 mM HEPES (pH 7.4) and 10 mM Glucose^71^. Intact TGs attached to the cranial base were carefully isolated on ice-cold EVB from *Avil*-Cre:*R26*-GCaMP6s mice at different stages of development (P0–P1, P4–P6, P14–P16 > P60). Following dissection, the cranial base was affixed to the detachable base of the imaging chamber using superglue. Henceforth, the organ was continuously perfused with EVB, followed by high potassium buffer (10–15 min). The buffers were bubbled with 95% O_2_ and 5% CO_2_. For pharmacological studies, EVB was perfused for 25 min, then one or two of the following drugs was perfused for 20 min: PPADS (100 µM, Tocris), Suramin (500 µM, Sigma), DNQX (100 µM, Sigma), D-APV (50 µM, Abcam), Gabazine (100 µM, Abcam), Atropine (15 µM, Sigma), Strychnine (5 µM, Abcam), EGTA (50 mM, Sigma). Thereafter, the drug was washed away by perfusion with EVB for 20–30 min, following which high potassium buffer was perfused for 10 min to evaluate organ viability. All the buffers were maintained at a stable temperature of 25 °C and perfused at a rate of ~ 2 ml/min. Time lapse images were acquired at 1 Hz frequency using an upright microscope (Olympus, BX61), a monochromatic sCMOS camera (TELEDYNE Photometrics, Prime BSI) and a 20× water immersion lens (Olympus). All TGs were used within 3 h of dissection.

### Ex vivo calcium image analysis and quantification

#### Preprocessing

To analyze the firing pattern of individual cells, we bleach-corrected (Bleach correction plugin) and motion-corrected (Turbo-Reg plugin) the time-lapse images using Fiji/ImageJ version 1.53f, to minimize the effect of photobleaching and fix the inadvertent small movements during image acquisition. Slices with large fluctuations were removed from analyses.

#### Region of Interest (ROI) Detection

To detect spontaneously active neurons in a unbiased manner when comparing between the different age-groups (Fig. 3), we used the EZcalcium toolbox of MATLAB^72^, which uses the algorithm CaImAn^73^ for automated ROI detection. We used the following settings for ROI detection with CaImAn: initialization (Greedy), search method (Ellipse), deconvolution (Constrained foofsis SPGL1), autoregression (Decay), merge threshold (0.95), fudge factor (0.95), spatial downsampling (1), temporal downsampling (1), temporal iteration (5). If some ROI was not detected by the software, it was added manually. For all the detected ROIs, a MATLAB data file was generated which contained the extracted fluorescence (dF/F) for each ROI along with a contour plot displaying their individual shape and location. Next, using the ROI refinement module of the EZcalcium toolbox, we manually inspected the individual traces and shapes of each of the ROIs to exclude the false positives (such as low activity or incomplete peaks). A contour plot was generated for the refined set of ROIs. For generating the potassium plots (Supplementary Fig. 2), 15 random ROIs from each of the four stages of development were manually selected using Fiji/Image J. Their fluorescent intensities were also calculated using Fiji/Image J and further calculations were performed in Microsoft Excel.

#### Peak detection and characterization

All the ROIs detected and refined using the EZcalcium toolbox were further analyzed using the PeakCaller script written in MATLAB^74^ to characterize peaks of the calcium transients in details. We modified its script to calculate the peak parameters from raw data instead of the detrended data. The raw data was smoothed using the Savitzky-Golay filter to remove the effect of background fluctuations. Peak parameters were automatically calculated based on the given settings: required rise % (20), required fall % (15), maximum lookback (30), maximum lookahead (25). The exponential moving average (2-sided) option was selected for calculating the underlying trendline. dF/F threshold was taken as mean + standard deviation. Based on these settings, individual peaks for each ROI were detected. ROIs with no detectable peaks were manually excluded from analysis. The script calculated the amplitude, rise time, decay time and total duration of each peak. It also generated raster plots (Fig. 3f) to summarize peak properties from all the detected ROIs. Any false positives were manually removed from analysis using Microsoft Excel. For generating the potassium plots (Supplementary Fig. 2), calculations were done using Microsoft Excel. Sliding window F_o_ was taken as the average fluorescent intensity across 60 frames, and dF/F_o_ defined as (F_t_-F_o_)/F_o_ was calculated accordingly. Firing threshold was taken as 10*SEM. A binary heatmap was generated where dF/F_o_ ≥ threshold was taken as 1, and dF/F_o_ < threshold was taken as 0. In accordance with the schematic shown in Fig. 2a, quantitative analysis was done as follows: from Images 1, 2, and 3 for Fig. 3b,d and e; Images 1 and 2 for Fig. 3g–k; from Image 3 for Fig. 2g–k and Fig. 4f–h; from Image 4 for Fig. 4d–e,h.

#### Correlation analysis

For correlation analysis (Fig. 2g–k), ROIs were manually selected from all the detected spontaneously firing neurons in a 10 min duration (Image 3) using Fiji/ImageJ. Their fluorescent intensities were imported from Fiji/Image J and further calculations were done on Microsoft Excel. Sliding window F_o_ was taken as the average fluorescent intensity across 60 frames, and dF/F_o_ was calculated accordingly. Firing threshold was taken as Mean + standard deviation and only the transients exceeding this threshold were used for further analysis. For all the ROI pairs, Pearson’s correlation coefficient was calculated using Prism GraphPad. A correlation matrix was constructed, and hierarchal clustering was performed using R studio. The coordinates of the ROIs were exported from Fiji/Image J, and the distance between all the possible ROI pairs were calculated on Microsoft Excel using the following formula: Distance between two ROIs = $\sqrt {\left( {x2 - x1} \right)^{2} + \left( {y2 - y1} \right)^{2} }$, where x1, y1 and x2, y2 are respective the coordinates of the two ROIs.

#### Movie

For Supplementary Movies 1–5, F_0_ was subtracted from the bleach corrected images. Playback is 50 times faster than the real time.

### Image analysis of *Avil*-nlsRFP mouse

Intact TGs attached to cranial base were isolated from the *Avil*-nlsRFP Tg3 mouse (PB, TS, TI, unpublished) at different stages of development (P0–P1, P4–P6, P14–P16, > P60). Following dissection, the cranial base was affixed to the detachable base of the imaging chamber using superglue. Henceforth, the organ was continuously perfused with EVB. For counting the total number of neuronal nuclei visible in each frame (Fig. 3c,d), images were taken with an upright microscope (Olympus, BX61), a monochromatic sCMOS camera (TELEDYNE Photometrics, Prime BSI) and a 20× water immersion lens (Olympus). Image analysis was performed using Fiji/ImageJ. Firstly, the background was subtracted (100) and the image was made binary. Then, it was converted to a mask and the watershed function was applied. Lastly, the particle size was analyzed to count the total number of neuronal nuclei. Low magnification images were acquired using the M205 FCA microscope (Leica) and DFC7000T camera (Leica).

### Temporal color-coding maps

For the pharmacological inhibitor experiments (Fig. 5), temporal color-coding maps were generated using Fiji/Image J. Firstly, the time-lapse image was bleach corrected and registered. Then an F_o_ image was created by taking the average of all the images in the stacks. This F_o_ image was subtracted from each of the individual images in the stack to produce an F − F_o_ time-lapse, and a gaussian filter was applied. This processed dF/F_o_ time lapse was used for making the temporal color-coding maps.

### RNA sequencing and analysis

#### RNA isolation and library construction

TGs were carefully isolated from P5 and P15 C57BL/6J mice on ice, and immediately transferred to ice-cold RNALater solution (Sigma) in which they were incubated at room temperature overnight. Next day, they were transferred to 4 °C where they were stored for up to 1 week. RNA was isolated using the RNEasy Mini Kit (Qiagen, #74104) and stored in − 80 °C. Quality of the isolated RNA was assessed with the 2100 Bioanalyzer using the RNA 6000 nano kit (Agilent Technologies), and only the samples having RNA integrity number (RIN) > 8 were used further. RNA seq library preparation was performed using the TruSeq stranded mRNA sample preparation kit (Illumina). The quantity and quality of the library was assessed using the Qubit BR kit (Invitrogen) and Bioanalyzer DNA 1000 kit, respectively (Agilent Technologies).

#### Sequence alignment and analysis

Paired end sequencing was performed using the HiSeq 2500 (Illumina) at a depth of 24 million reads/sample. The quality control for the sequence reads was conducted by the Trimmomatic^75^. After trimming the adaptor sequences, we excluded the reads having the average base quality score < 25. The low-quality bases (base quality < 20) at the head and tail of each read were trimmed. We excluded the reads if the lengths of the reads were < 50 after the trimming. Then, the processed reads were aligned to the mouse genome (mm 10) using GSNAP^76^. The sequence alignment map (SAM) files were converted to the binary alignment map (BAM) files by using the SAMtools^77^. The number of reads aligning to each gene were counted using featureCount^78^, and the raw expression counts were converted to counts per million (CPM). After adding the offset of 1, the CPM values were converted to the log-2 scale. The Limma/voom pipeline was used to detect differentially expressed genes between P5 TG and P15 TG^79^. Statistical significance was assessed by False discovery rate (FDR).

### Quantitative RT-PCR

The RNA samples that were used for RNA sequencing were also utilized for performing quantitative RT-PCR (qRT-PCR). cDNA was synthesized using the PrimeScript RT reagent kit (Takara, #RR037Q), and was used as templates for qRT-PCR in 1:100 dilution. 2X SYBR Green (Applied Biosystems, #4367669) was used for the enzymatic reaction and real-time PCR was performed (Takara thermocycler: TP970 and TP850). Primer pairs OP11/OP12, OP19/OP20, and OP29/OP30 were used for *GAPDH*, *P2RX3*, and *Avil*, respectively. The sequence for all the primers is mentioned in Supplementary Table 3. Each sample had 2 biological replicates and 2 technical replicates.

### Statistical Analysis and Computing

Fiji/ImageJ ver. 1.53f51^80^, MATLAB (2021) with Image Processing, Parallel Computing, Signal Processing, and Statistics and Machine Learning toolboxes (Mathworks), Microsoft Excel, Rstudio version 2021.09.2, and Prism GraphPad version 9.3.1 were used for data analysis and visualization. Unless otherwise mentioned, data are presented as mean ± standard error of mean (SEM). Statistical analyses were performed using Prism GraphPad. Shapiro–Wilk test was performed to check for data normality. *p* value < 0.05 was considered significant. The asterisks in the figures indicate as follows: * for *p* < 0.05, ** for *p* < 0.005, *** for *p* < 0.001, and **** for *p* < 0.0001. For the violin plot (Fig. 2k), the middle dashed horizontal line represents the median, and the two dotted horizontal lines represent the quartiles. Sample size for all the results is described in the figure legends.

## Supplementary Information

Supplementary Information 1.

Supplementary Video 1.

## Data Availability

The RNA sequencing datasets generated and analyzed during the current study are available in the DNA Data Bank of Japan (DDBJ) repository (PRJDB13784). The codes used for analysis and the data that support the findings are available from the authors (P.B., T.I.) upon reasonable request.
