# Supplementary material for: Spontaneous activity in whisker-innervating region of neonatal mouse trigeminal ganglion
Source: Sci Rep. 2022 Sep 29;12:16311. doi: 10.1038/s41598-022-20068-z (PMC9522796; doi:10.1038/s41598-022-20068-z)
Supplement: Supplementary file 1 — Supplementary Information 1. [file 41598_2022_20068_MOESM1_ESM.pdf]

# Spontaneous Activity in Whisker-Innervating Region of Neonatal Mouse Trigeminal Ganglion

Piu Banerjee, Fumi Kubo, Hirofumi Nakaoka, Rieko Ajima, Takuya Sato, Tatsumi Hirata, Takuji Iwasato*

**Supplementary Data File**

Total: 11 pages

Supplementary Figures: 5

Supplementary Tables: 3

Supplementary Movies (titles): 5

**Supplementary Table 1: Candidate genes expression in developing TG by RNA Sequencing**

| Category | Gene | P5 TG | P15 TG | Log-2 Fold Change | adj. P. Value |
|---|---|---|---|---|---|
| Purinergic Receptors | *P2RX1* | 0.84 | 0.67 | 0.24 | 4.94E-01 |
| | *P2RX2* | 4.07 | 2.31 | 0.55 | 1.59E-04 |
| | *P2RX3* | 486.68 | 180.45 | 1.45 | 4.84E-13 |
| | *P2RX4* | 27.13 | 33.58 | -0.41 | 5.63E-07 |
| | *P2RX5* | 5.73 | 15.18 | -1.49 | 2.54E-11 |
| | *P2RX6* | 6.32 | 31.07 | -2.37 | 4.56E-13 |
| | *P2RX7* | 58.75 | 47.94 | 0.18 | 1.51E-01 |
| | *P2RY1* | 44.52 | 51.06 | -0.29 | 9.84E-04 |
| | *P2RY2* | 27.57 | 28.50 | -0.17 | 8.59E-02 |
| | *P2RY6* | 3.65 | 3.45 | -0.13 | 3.45E-01 |
| | *P2RY12* | 16.70 | 18.48 | -0.25 | 1.50E-02 |
| | *P2RY13* | 3.01 | 3.37 | -0.32 | 1.74E-02 |
| | *P2RY14* | 4.34 | 5.74 | -0.54 | 3.76E-05 |
| Glutamatergic Receptors | *GRIA1* | 20.70 | 19.36 | -0.01 | 8.53E-01 |
| | *GRIA2* | 59.99 | 40.19 | 0.49 | 1.64E-06 |
| | *GRIA3* | 31.88 | 30.73 | -0.05 | 3.65E-01 |
| | *GRIA4* | 76.48 | 86.87 | -0.25 | 1.32E-03 |
| | *GRID1* | 20.27 | 20.49 | -0.12 | 5.95E-02 |
| | *GRID2* | 22.44 | 10.47 | 0.95 | 9.52E-08 |
| | *GRID2ip* | 3.10 | 3.67 | -0.37 | 1.37E-02 |
| | *GRIK1* | 283.55 | 162.17 | 0.78 | 2.91E-09 |
| | *GRIK2* | 22.42 | 13.73 | 0.56 | 7.33E-06 |
| | *GRIK3* | 47.33 | 30.92 | 0.50 | 6.63E-06 |
| | *GRIK4* | 42.95 | 40.02 | 0.01 | 8.94E-01 |
| | *GRIK5* | 80.33 | 44.80 | 0.76 | 9.58E-10 |
| | *GRIN1* | 168.55 | 121.70 | 0.42 | 5.33E-06 |
| | *GRINA* | 232.79 | 323.65 | -0.50 | 1.47E-07 |
| | *GRIN2A* | 1.44 | 0.36 | 1.14 | 4.60E-06 |
| | *GRIN2B* | 2.28 | 0.71 | 1.03 | 5.19E-04 |
| | *GRIN2C* | 1.34 | 1.39 | -0.12 | 3.79E-01 |
| | *GRIN2D* | 7.63 | 2.56 | 1.27 | 1.29E-08 |
| | *GRIN3A* | 33.94 | 20.81 | 0.59 | 4.88E-08 |
| | *GRM1* | 1.27 | 0.85 | 0.24 | 4.70E-01 |
| | *GRM2* | 1.27 | 1.55 | -0.33 | 5.77E-02 |
| | *GRM3* | 4.57 | 1.49 | 1.20 | 2.39E-08 |
| | *GRM4* | 35.70 | 46.00 | -0.46 | 1.31E-05 |
| | *GRM5* | 5.64 | 2.72 | 0.70 | 7.09E-03 |
| | *GRM7* | 41.42 | 30.37 | 0.34 | 1.12E-03 |
| | *GRM8* | 16.97 | 17.80 | -0.18 | 1.71E-02 |
| GABAergic Receptors | *GABRA1* | 61.31 | 166.36 | -1.50 | 3.45E-12 |
| | *GABRA2* | 31.67 | 47.50 | -0.67 | 2.38E-06 |
| | *GABRA3* | 18.71 | 13.71 | 0.31 | 3.00E-03 |
| | *GABRA4* | 8.60 | 6.24 | 0.27 | 1.93E-02 |
| | *GABRA5* | 33.55 | 24.82 | 0.31 | 2.05E-03 |
| | *GABRB1* | 13.92 | 9.14 | 0.46 | 6.53E-04 |
| | *GABRB2* | 15.58 | 20.28 | -0.48 | 5.21E-04 |
| | *GABRB3* | 147.94 | 109.36 | 0.38 | 2.92E-06 |
| | *GABRD* | 1.14 | 2.01 | -0.69 | 3.75E-04 |
| | *GABRG1* | 35.95 | 57.48 | -0.76 | 5.49E-08 |
| | *GABRG2* | 112.62 | 121.17 | -0.15 | 2.12E-02 |

| | | | | | |
|---|---|---|---|---|---|
| | *GABRG3* | 5.87 | 1.32 | 1.69 | 6.53E-09 |
| | *GABBR1* | 410.52 | 312.15 | 0.40 | 5.42E-07 |
| | *GABBR2* | 305.62 | 227.72 | 0.39 | 3.79E-06 |
| Cholinergic Receptors | *CHRM1* | 7.96 | 2.75 | 1.23 | 2.27E-08 |
| | *CHRM2* | 26.44 | 21.65 | 0.16 | 1.39E-01 |
| | *CHRM3* | 5.94 | 4.32 | 0.23 | 4.66E-02 |
| | *CHRM4* | 8.70 | 4.25 | 0.79 | 6.48E-08 |
| | *CHRNA3* | 4.23 | 5.56 | -0.54 | 8.17E-06 |
| | *CHRNA4* | 10.53 | 3.18 | 1.44 | 3.52E-09 |
| | *CHRNA5* | 1.02 | 0.51 | 0.53 | 2.09E-03 |
| | *CHRNA6* | 6.71 | 32.02 | -2.32 | 1.56E-12 |
| | *CHRNA7* | 21.14 | 17.89 | 0.12 | 3.05E-01 |
| | *CHRNB1* | 1.23 | 1.45 | -0.25 | 1.31E-01 |
| | *CHRNB2* | 70.15 | 85.68 | -0.37 | 5.14E-06 |
| | *CHRNB3* | 6.61 | 12.52 | -1.04 | 4.29E-08 |
| | *CHRNB4* | 2.49 | 2.98 | -0.46 | 2.01E-02 |
| Glycinergic Receptors | *GLRA2* | 5.10 | 1.55 | 1.32 | 9.65E-08 |
| | *GLRB* | 98.08 | 198.76 | -1.06 | 1.14E-11 |

**Supplementary Table 2: Calcium channels expression in developing TG by RNA Sequencing.**

| Categories | Gene | P5 TG | P15 TG | Log-2 Fold Change | adj. P. Value |
|---|---|---|---|---|---|
| Cation-Channel Sperm Associated | *CATSPERD* | 0.94 | 0.94 | -0.08 | 6.16E-01 |
| | *CATSPERZ* | 9.18 | 8.79 | -0.07 | 4.51E-01 |
| | *CATSPER2* | 2.66 | 4.08 | -0.69 | 3.38E-06 |
| Two-Pore Segment Channel | *TPCN1* | 61.91 | 67.75 | -0.21 | 4.89E-04 |
| | *TPCN2* | 7.64 | 4.31 | 0.59 | 1.36E-06 |
| Voltage-Gated Calcium Channel | *CACNG2* | 10.10 | 17.97 | -0.92 | 2.21E-09 |
| | *CACNG3* | 8.39 | 3.07 | 1.16 | 3.09E-09 |
| | *CACNG4* | 28.16 | 20.57 | 0.33 | 8.02E-04 |
| | *CACNG5* | 86.25 | 57.05 | 0.50 | 9.26E-07 |
| | *CACNG7* | 12.61 | 16.29 | -0.48 | 2.29E-05 |
| | *CACNA1A* | 108.25 | 85.68 | 0.26 | 3.64E-03 |
| | *CACNA1B* | 172.76 | 67.18 | 1.29 | 1.83E-10 |
| | *CACNA1C* | 55.09 | 29.83 | 0.77 | 9.94E-08 |
| | *CACNA1D* | 18.42 | 11.26 | 0.56 | 8.74E-06 |
| | *CACNA1E* | 8.81 | 5.15 | 0.55 | 1.35E-04 |
| | *CACNA1G* | 21.68 | 6.01 | 1.63 | 1.30E-05 |
| | *CACNA1H* | 76.81 | 38.30 | 0.90 | 4.94E-09 |
| | *CACNA1I* | 4.70 | 4.79 | -0.21 | 3.58E-02 |
| | *CACNA1S* | 0.84 | 1.06 | -0.30 | 4.35E-02 |
| | *CACNA2D1* | 145.50 | 90.85 | 0.62 | 1.33E-06 |
| | *CACNA2D2* | 77.05 | 32.46 | 1.14 | 1.91E-11 |
| | *CACNA2D3* | 55.66 | 76.95 | -0.55 | 1.29E-06 |
| | *CACNB1* | 68.28 | 45.41 | 0.50 | 5.73E-07 |
| | *CACNB2* | 5.89 | 7.69 | -0.52 | 1.74E-04 |
| | *CACNB3* | 244.93 | 240.76 | 0.00 | 9.67E-01 |
| | *CACNB4* | 111.57 | 138.06 | -0.36 | 5.06E-05 |
| Ryanodine Receptors | *RYR1* | 2.15 | 1.16 | 0.59 | 4.65E-04 |
| | *RYR2* | 29.48 | 29.36 | -0.12 | 3.64E-01 |
| | *RYR3* | 11.30 | 8.73 | 0.22 | 5.60E-02 |
| Inositol Triphosphate Receptors | *ITPR1* | 18.64 | 24.89 | -0.52 | 1.21E-07 |
| | *ITPR2* | 13.17 | 8.68 | 0.40 | 2.12E-02 |
| | *ITPR3* | 182.17 | 145.56 | 0.25 | 6.45E-02 |

**Supplementary Table 3: Primer Sequences**

| Primer ID | Primer Sequence (5' to 3') |
|---|---|
| KS157 (*Avil* Cre) | CCCTGTTCACTGTGAGTAGG |
| KS158 (*Avil* Cre) | AGTATCTGGTAGGTGCTTCCAG |
| KS159 (*Avil* Cre) | TGTTTCACTATCCAGGTTACGGA |
| KS109 (GCaMP6s) | GTGGACTCATCACGTCGTAAGTGGAATAAG |
| KS110 (GCaMP6s) | CCAGGGCACGGACAGCTTGCCGGTGG |
| KS149 (*Avil*-nlsRFP) | GGCACCCAGACCATGAAGAT |
| KS150 (*Avil*-nlsRFP) | CCAGTTTGCTAGGGAGGTCG |
| KS253 (*P2RX*3) | TAGCACCGGAGTGCATCTTG |
| KS254 (*P2RX*3) | GCAACCCCTAGCATGTGTCT |
| OP31 (*P2RX*3) | CGATCCATTCCGATCGTCCC |
| OP32 (*P2RX*3) | GATGAGAGGGCAGCCAAGAG |
| OP11 (*GAPDH*) | CACAATTTCCATCCCAGACC |
| OP12 (*GAPDH*) | GTGGGTGCAGCGAACTTTAT |
| OP19 (*P2RX3*) | CTTCCGTGGGAGTGGGGACTGTT |
| OP20 (*P2RX3*) | GCCCCCGAGTCTGTGGACTGC |
| OP29 (*Avil*) | GGTCAGTTCCAGGAAGACAG |
| OP30 (*Avil*) | CTCGTAGAAGTTGCCGTGAG |
| sgRNA_62Forw | TATTAATGCAGCACGTTTCT |
| sgRNA_1228Forw | CTCCCATCTAGACCCGTCTC |

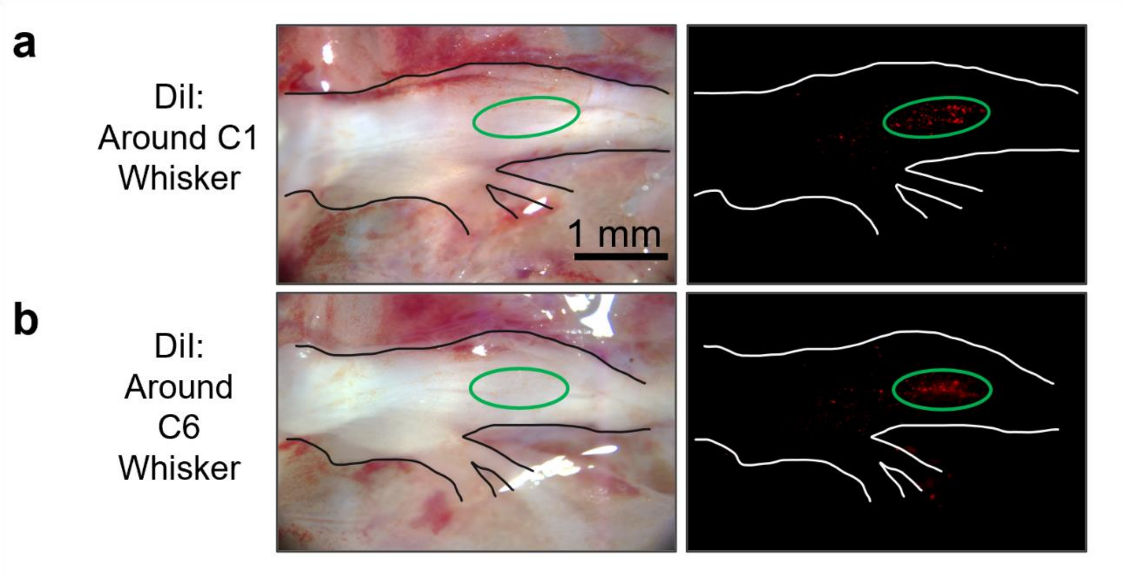


**Supplementary Figure 1:** Labeled neuron localization in P5 TG 5 days after placing DiI crystals around C1 whisker (N= 3) (a), and around C6 whisker (N= 3). Brightfield image (Left) and RFP filter image (Right) are shown. Green circles enclose the region of labeled neuron localization.

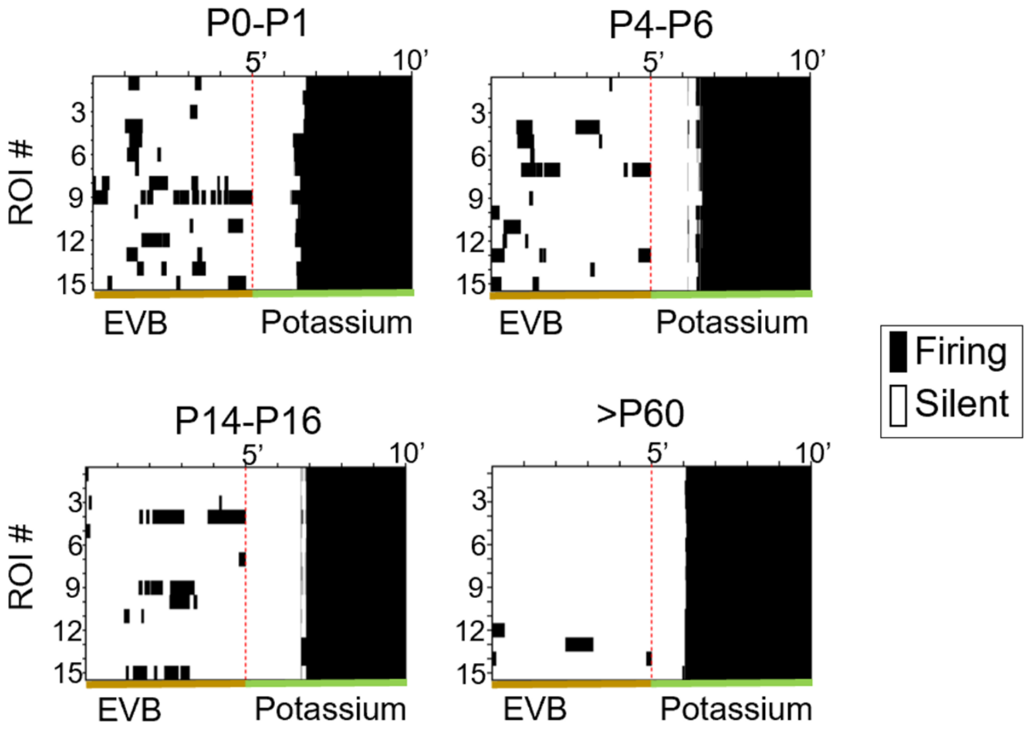


**Supplementary Figure 2:** Binary raster plots showing the reaction of ROIs to high potassium buffer. Only the calcium transients exceeding the threshold are counted as firing events. (Firing threshold: 10*SEM). Fifteen neurons were randomly selected from an animal per age group. [N= 2 animals in each stage].

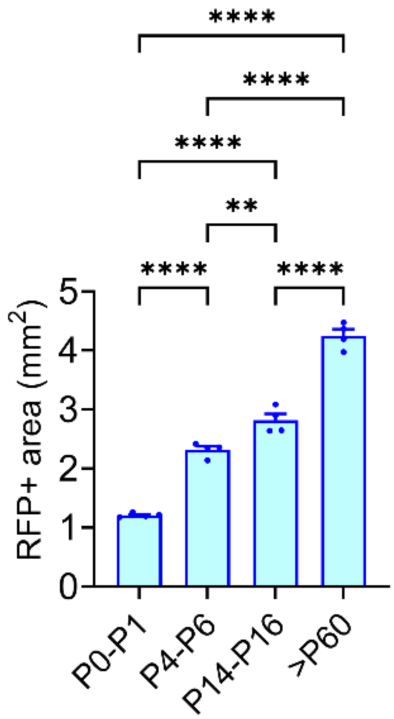


**Supplementary Figure 3:** Bar graph representing the size of the RFP$^+$ area in the intact TG of *Avil*-nlsRFP mouse across the four stages of development (Avg. of L1, L2, and L3). Each circle signifies an individual TG. One-way ANOVA with Tukey's multiple comparison test is performed. [P0–P1 (n= 4 animals), P4–P6 (n= 6), P14–P16 (n= 6), >P60 (n= 4)].

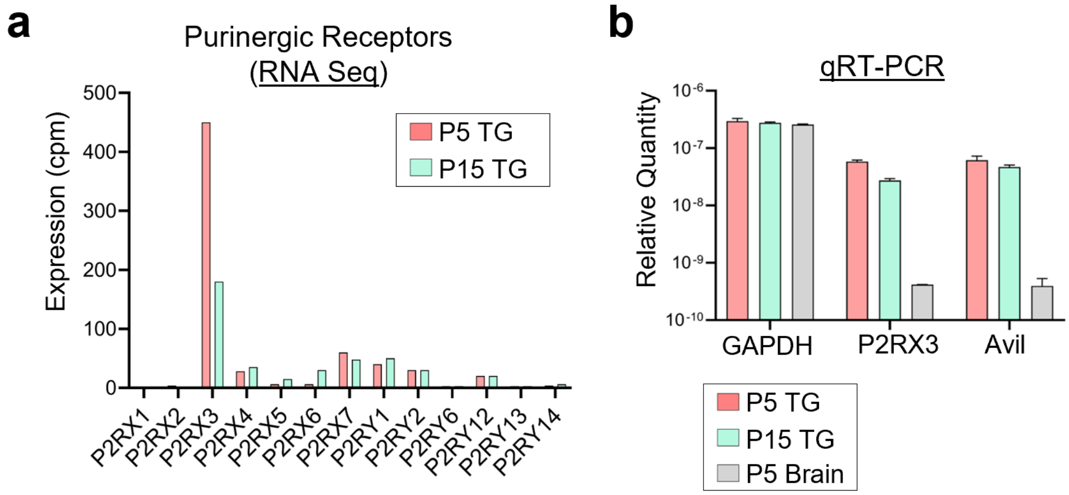


**Supplementary Figure 4: Expression of *P2RX3* in the developing TG**

a) Expression of all the known purinergic receptors in P5 TG and P15 TG analyzed by RNA sequencing. The expression values are given in counts per million (cpm). b) Expression of *P2RX3* gene in P5 TG, P15 TG, and P5 brain analyzed by qRT-PCR. GAPDH is used as a housekeeping gene and *Avil* (Advillin) is used as a sensory neuron-specific gene (n= 2 each) (Error bar: SEM).

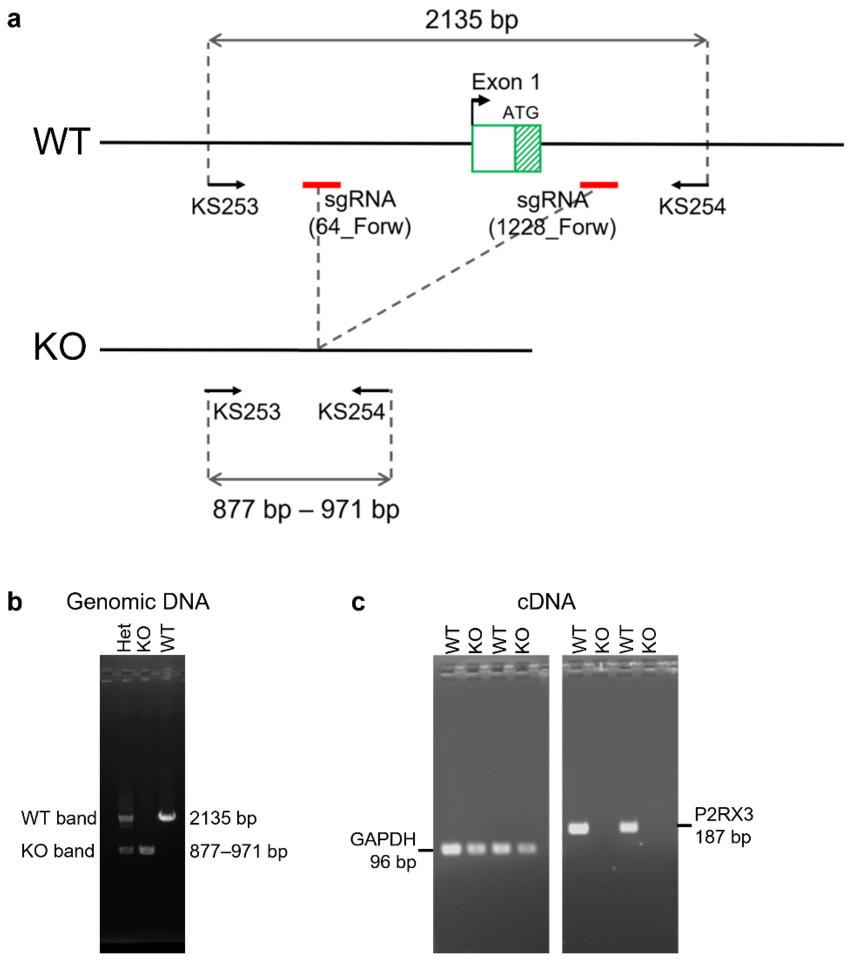


**Supplementary Figure 5: Generation of *P2RX3* KO mice**

a) Schematic describing the construction design for *P2RX3* KO mouse using CRISPR/Cas9. Exon 1 (307 bp) of the *P2RX3* gene is flanked by two designed single guide RNAs (sgRNAs) (64_Forw, 1228_forw) to target that area for deletion. ATG is the translation initiation codon. Location of genotyping primers (KS253/254) are shown. b) Representative gel image showing genotyping results for *P2RX3*$^{+/+}$ (WT), *P2RX3*$^{-/-}$ (KO), and *P2RX3*$^{+/-}$ (Het) littermates by PCR with primers (KS253/254). c) Validation of gene knockout in *P2RX3*$^{-/-}$ animals by RT-PCR with primers for exons 10-12 (OP19/OP20). TG of P4–P6 *P2RX3*$^{-/-}$ (KO) and wild-type (WT) littermates were used. *GAPDH* was used as a housekeeping gene.

**SUPPLEMENTARY MOVIE LEGENDS:**

Movie 1:

Spontaneous activity in the TG of *Avil*-Cre:*R26*-GCaMP6s mouse at P5 (5 min).

Movie 2:

Spontaneous activity in the TG of *Avil*-Cre:*R26*-GCaMP6s mouse at P0 (5 min).

Movie 3:

Spontaneous activity in the TG of *Avil*-Cre:*R26*-GCaMP6s mouse at P15 (5 min).

Movie 4:

Spontaneous activity in the TG of *Avil*-Cre:*R26*-GCaMP6s mouse in adulthood (5 min).

Movie 5:

Effect of EGTA (50 mM) application on spontaneous activity in the TG of *Avil*-Cre:*R26*-GCaMP6s mouse at P5.
